# Protective effects of activated vitamin D receptor on radiation‐induced intestinal injury

**DOI:** 10.1111/jcmm.17645

**Published:** 2022-12-29

**Authors:** Yuhan Lin, Penglin Xia, Fangyu Cao, Cheng Zhang, Yajie Yang, Haitao Jiang, Haishan Lin, Hu Liu, Ruling Liu, Xiaodong Liu, Jianming Cai

**Affiliations:** ^1^ School of Public Health and Management Wenzhou Medical University Zhejiang China; ^2^ Department of Radiation Medicine, Faculty of Naval Medicine Naval Military Medical University Shanghai China; ^3^ Incubation Base for Undergraduates' Innovative Practice in Department of Radiation Medicine, Faculty of Naval Medicine Naval Military Medical University Shanghai China; ^4^ Department of Oral and maxillofacial Trauma and Orthognathic Surgery Stomatological Hospital of Zunyi Medical University Zunyi China; ^5^ Cancer Centre, Beijing Friendship Hospital Capital Medical University Beijing China

**Keywords:** HIF/PDK1, radiation protection, radiation‐induced intestinal injury, tumour radiotherapy, vitamin D

## Abstract

Radiation‐induced intestinal injury (RIII) is a common complication after radiation therapy in patients with pelvic, abdominal, or retroperitoneal tumours. Recently, in the model of DSS (Dextran Sulfate Sodium Salt) ‐induced intestinal inflammatory injury, it has been found in the study that transgenic mice expressing hVDR in IEC (Intestinal Epithelial Cell) manifest highly anti‐injury properties in colitis, suggesting that activated VDR in the epithelial cells of intestine may inhibit colitis by protecting the mucosal epithelial barrier. In this study, we investigated the effect of the expression and regulation of VDR on the protection of RIII, and the radiosensitivity in vitro experiments, and explored the initial mechanism of VDR in regulating radiosensitivity of IEC. As a result, we found that the expression of VDR in intestinal tissues and cells in mice can be induced by ionizing radiation. VDR agonists are able to prolong the average survival time of mice after radiation and reduce the radiation‐induced intestinal injury. For lack of vitamin D, the radiosensitivity of intestinal epithelial cells in mice increased, which can be reduced by VDR activation. Ensuing VDR activation, the radiation‐induced intestinal stem cells damage is decreased, and the regeneration and differentiation of intestinal stem cells is promoted as well. Finally, on the basis of sequencing analysis, we validated and found that VDR may target the HIF/PDK1 pathway to mitigate RIII. We concluded that agonism or upregulation of VDR expression attenuates radiation‐induced intestinal damage in mice and promotes the repair of epithelial damage in intestinal stem cells.

## 1 INTRODUCTION

Radiation‐induced intestinal injury is the leading cause resulting in early death of the wounded in nuclear and radiation emergencies, as well as a common complication after radiation therapy in patients with clinical superior pelvic, intraperitoneal, or retroperitoneal tumours.^1^, ^2^, ^3^, ^4^ When intestinal tissue is exposed to ionizing radiation, biological macromolecules, such as DNA and proteins, were damaged, further leading to cell metabolic disorder, changes in cellular structure and eventually, the cell death.^5^, ^6^ On the other hand, unlike other tissues and organs, with the continuous renewal and differentiation of their stem cells, the intestinal tissues maintain the integrity of the intestinal epithelial structure.^7^, ^8^ Involving various aspects, such as cell death, stem cell damage, immune activation, nutriregulation disorders and microcirculation disorders, the mechanism of radiation‐induced intestinal damage is considerably complicated. With no ideal treatment at home and abroad so far, the mortality rate is almost 100% once damage occurs.^9^ Therefore, it is of great significance to study the prevention and treatment of radiation‐induced intestinal damage.^10^

Recently, in the model of DSS (Dextran Sulfate Sodium Salt) ‐induced intestinal inflammatory injury, it has been found in the study that transgenic mice expressing hVDR in IEC (Intestinal Epithelial Cell) manifest highly anti‐injury properties in colitis, suggesting that activated VDR in intestinal epithelial cells may inhibit colitis by protecting the mucosal epithelial barrier.^11^ Maintaining the integrated functional mucosal barrier which inhibits colitis, VDR thus serves as the main defence mechanism against colitis, and in turn prevents the interaction between antigens and bacteria in intestinal lumen and immune components in the intestinal lamina propria.^12^ It has been indicated that mucosal recovery and the migration of epithelial cells is accompanied with the upregulation of VDR expression after intestinal damage, suggesting the vital role VDR plays during the procedure of intestinal damage repair. There were few relevant reports about researches on relations between VDR and radiation‐induced intestinal injury so far. Meanwhile, other studies on VDR and radiation‐induced damage are also limited to UV irradiation,^13^ lacking reports corresponding ionizing radiation‐induced damage.

In this study, we revealed that IR can enhance the VDR expression in mice, and the activation of VDR can alleviate the radiation‐induced intestinal injury in mice, prolong the average survival time, and promote proliferation and regeneration of intestinal stem cells by targeting HIF/PDK1.

## 2 MATERIAL AND METHOD

### 2.1 Animals

Wild‐type male BALB/c mice were purchased from Shanghai Jihui Laboratory Animal Breeding Co., Ltd. Experimental mice were kept in standard animal laboratory, all the protocols approved by the Animal Ethics of Naval Military Medical University. Food and water were supplied ad libitum, with 12–12 h/light–dark cycle.

### 2.2 Major materials and reagents

CCK‐8 kit was purchased from Dongren Chemical Shanghai Co., Ltd (No. CK04). 1640 medium from Hyclone Co., Ltd (No. SH30809.01B), Annexin V‐FITC apoptosis kit from TransGen Biotech (No. FA101‐02); Edu kit were bought from Beyotime Co., Ltd. Anti‐OLFM4 antibody (39141), Anti‐c‐myc antibody (13987), Anti‐Yap1 antibody (14074), Anti‐LRP6 antibody (3395), and Anti‐Naked2 antibody (2596) were purchased from CST Co., Ltd; Anti‐Axin1 antibody (ab300134), Anti‐β‐catenin antibody (ab223075), Anti‐Wnt3a antibody (ab219412) were purchased from Abcam Co., Ltd. BX‐795, the inhibitor of PDK1 and PT2385, the inhibitor of HIF2 were purchased from Selleck Co., Ltd (No. S1274 and S8352).

### 2.3 Establishment of Radiation‐Induced Intestinal Injury Model of Mice

Radiation was delivered by the ^60^Co (Cobalt) source in the Irradiation Center of the Naval Military Medical University, with doses depending on the experimental requirements. The dose of abdominal irradiation was 25 Gy at a dose rate of 1 Gy/min, while the dose of total body irradiation was 7 Gy at a dose rate of 1 Gy/min.

### 2.4 Cell culture

The intestinal epithelial cells (MODE‐K) derived from mice belong to adherent cells. MODE‐K cells (donated by Shanghai Institutes for Biological Sciences) were grown at 37°Cwith 5% CO_2_ in RPMI Medium supplemented with 10% FBS and 1% antibiotics. Cellular state and density were observed during culture and fluid was changed on alternate days.

### 2.5 Apoptosis assays

Annexin V‐FITC/PI Cell Apoptosis Detection Kit (TransGen Biotech Corp., Ltd) was used for apoptosis analysis of wild‐type cell lines. Briefly, cells were digested with EDTA‐free trypsin, harvested and resuspended in 100ul Annexin V‐FITC binding buffer. Added with 5 μl Annexin V‐FITC and Propidium iodide respectively, binding buffer was gently mixed and incubated for 30 min. Subsequently, apoptosis was analysed by flow cytometry.

### 2.6 Cell cycle

Processed cells were stained by Cell cycle detection kit (BD biosciences), and then cell cycle was analysed by flow cytometry.

### 2.7 Detection of cell proliferation

At 48 h after irradiation, cells were further incubated for 2 h in 6‐well plates with the same amount of 20 μM of Edu in each well, which has been prepared by use of cell culture medium and preheated to 37°C. Removed of the medium after inubation, cells subsequently underwent digestion, 4% paraformaldehyde fixation, 0.3% Triton reaction buffer perforation and centrifugation. Ensuing the removal of supernatant, cells were added with 0.5 ml Click fixative and incubated for 30 min at room temperature, protected from light. Cells were then centrifuged, removed of the reaction fluid, washed for three times with cleaning solution and went through centrifugation again. Subsequently, cells were resuspended with PBS and detected by flow cytometry.

### 2.8 Histopathology and immunohistochemistry

Small intestinal tissues of BALB/c mice were fixed using 4% paraformaldehyde for 24 h. Tissues were embedded in paraffin and sectioned at a thickness of 6 μm using microtome. Sections were stained with haematoxylin and eosin, sealed after dehydration with absolute ethanol, and then observed and photographed using a microscope. After dewaxing and hydration, sections went through antigen retrieval in high‐pressure method, rinse, sealing and primary antibody incubation. Sections subsequently underwent rinse, secondary antibody incubation, chromogenic reaction, counterstain and were finally observed under the microscope.

### 2.9 Extraction of intestinal organoids

The intestinal organoids of BALB/c mice were extracted in the sterile environment, shaked on ice for 1 h and oscillated 3 times at 3 min per time. Then, the vortexed intestinal suspension was passed through a 70 μm filter and collected to centrifuge at 290 rpm for 5 min. After discarding the supernatant, the filtrate precipitate was washed with PBS (this step was repeated three times). After mixing the medium (STEMCELL Technologies) to matrigel (Corning company) in a 1:1 ratio, precipitate was inoculated into a 24‐well plate in a volume of 50 μl, let stand in the incubators for 30 min, added with 0.5 ml/well of medium, and subsequently cultured in the incubators for observation.

### 2.10 Western blot analysis

After being extracted under the instruction of cytoprotein extraction method and tissue protein extraction method from the acquired relevant samples, proteins were loaded in 15% SDS‐PAGE gel (Epizyme Biotech), transferred onto the membrane following electrophoresis. Ensuing the transfer, membranes were blocked with 5% nonfat milk for 2 h, washed with TBST at intervals of 10 min for three times at 4°C and then incubated in primary specific antibody. Subsequently, the membranes were washed in TBS containing 0.1% Tween‐20 (TBST) for three times and incubated in secondary antibody at room temperature for 75 min. After being washed in TBST for three times again, the membranes went through exposure using HRP chromogen substrates under the E‐Gel Imager.

### 2.11 Statistical analysis

The experimental data were expressed as mean ± SD and analysed by SPSS19.0 software. T test was applied to detect differences between two groups (consistent with normal distribution; Unpaired *t*‐test for equal variance and unpaired *t*‐test with Welch's correction test for unequal variance). We used an analysis of variance (anova) test (with normal distribution, equal variance) to analyse differences among over three groups. Correlation analysis was performed via rank‐sum test. *p* < 0.05 was considered as statistically significant.

## 3 RESULTS

### 3.1 Ionizing radiation can induce an upregulation of VDR expression in mouse intestinal tissues and cells

**FIGURE 1:**
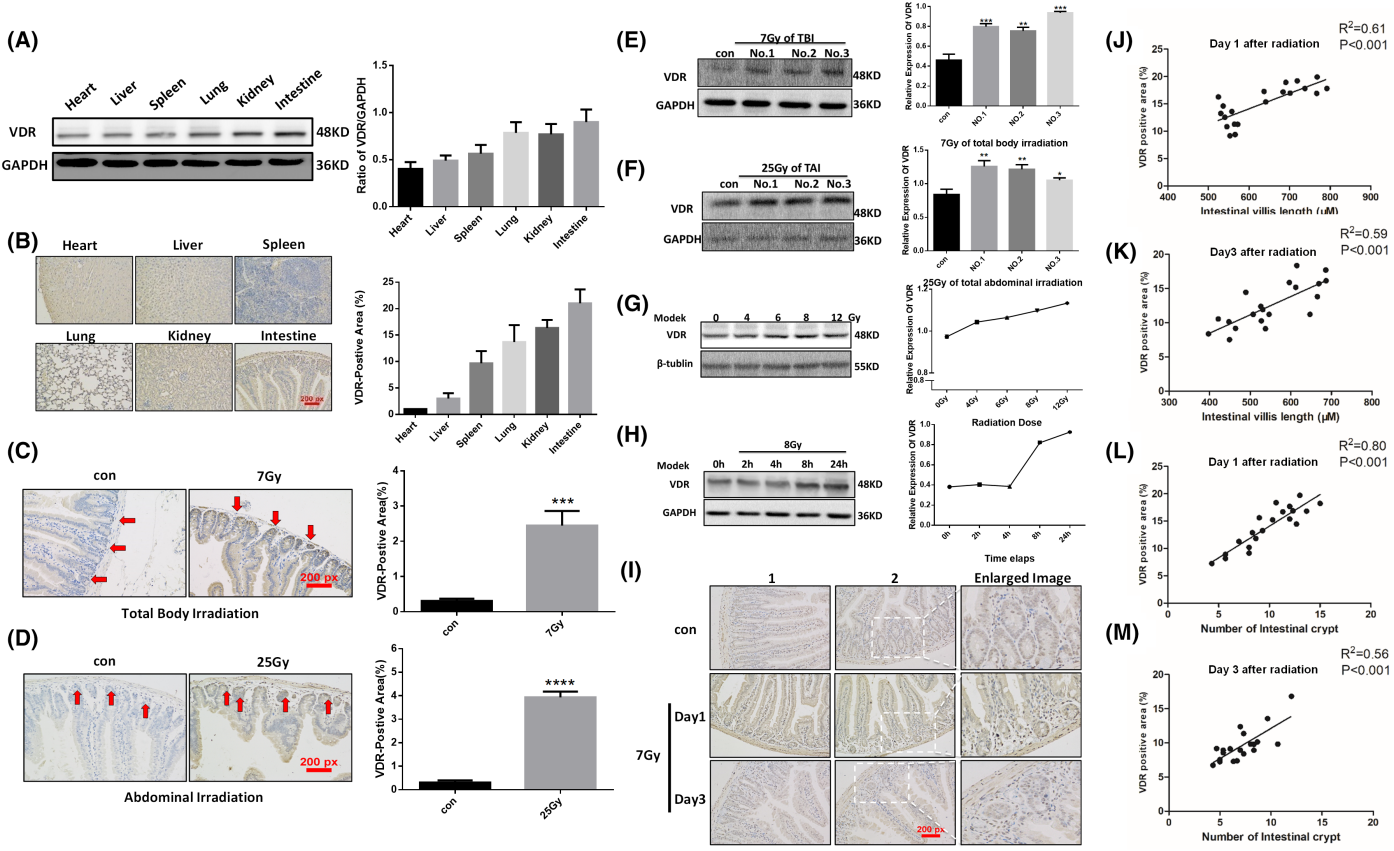
Effects of ionizing radiation on VDR expression in intestinal tissues and epithelial cells of mice. (A) VDR expression in various organs and tissues of mice was testified by Western Blot Analysis. (B) VDR expression in various organs of mice was detected by immunohistochemistry. (C) Immunohistochemical staining of VDR in intestinal tracts of mice after 7 Gy of general irradiation. (D) Immunohistochemical staining of VDR in intestinal tracts of mice after 25 Gy of abdominal irradiation. (E) VDR in intestinal tracts of mice was detected by Western Blot Analysis after 7 Gy of general irradiation. (F) VDR in intestinal tracts of mice was detected by Western Blot Analysis after 25 Gy of abdominal irradiation. Con stands for non‐irradiated group and the others are irradiated groups. No. 1–3 represented three separate samples. G. Radiation‐induced VDR expression in Modek cells was dose‐dependent. (H) VDR expression induced by 8 Gy irradiation in Modek cells was time‐dependent. (I) VDR immunohistochemical map. (J) The relationship between intestinal villus length and VDR expression on day 1 after irradiation. (K) The relationship between intestinal villus length and VDR expression on day 3 after irradiation. (L) The relationship between intestinal crypt number and VDR expression on day 1 after irradiation. (M) The relationship between intestinal crypt number and VDR expression on day 3 after irradiation. The statistical results are expressed by Mean ± SD, *** and **** represents *p* < 0.001 and *p* < 0.0001, respectively. Each experiment was repeated for three times. *N* = 7

Aiming to explore the relationship between ionizing radiation and VDR expression in mouse intestinal tissues, we firstly need to clarify the VDR expression in intestinal tissues. It can be indicated in experiments (Figure 1A,B), that VDR expression varies in different organs and tissues, among which expression in intestinal tissues is most obvious. On this basis, systemic irradiation and local abdominal irradiation was delivered to mice. It (Figure 1C–F, showed that both systemic irradiation and local abdominal irradiation upregulated the VDR expression of intestinal tissue, and especially, expression at the intestinal crypt was most obvious. Subsequently, MODE‐K cells taken as research object, it was demonstrated that (Figure 1G) VDR expression in MODE‐K cells increased with the rising irradiation dose. Extracting cytoproteins respectively at 0, 2, 4, 8, 24 h post irradiation with a dose of 8 Gy, we found that VDR expression in MODE‐K cells was continuously upregulated over time after 8 Gy irradiation (Figure 1H). It proved that ionizing radiation can directly induce VDR expression in Modek cells with dose and time dependent manner.

### 3.2 There is correlation between VDR expression and the extent of radiation‐induced intestinal damage in mice

It has been verified in previous experiments that irradiation can induce intestinal VDR expression in mice. Moreover, whether the occurrence and severity of radiation‐induced intestinal injury was correlated with VDR expression is still in need of investigation. Mouse intestinal immunohistochemical staining (Figure 1I) showed that the VDR expression in intestinal tissue increased, and the length of intestinal villus and the number of intestinal crypts was positively correlated with VDR expression (Figure 1J–M, *p*<0.001), indicating the negative correlation between VDR expression and the extent of intestinal damage.

### 3.3 VDR agonists extend the mean photographic survival of mice and attenuate radiation‐induced intestinal injury

**FIGURE 2:**
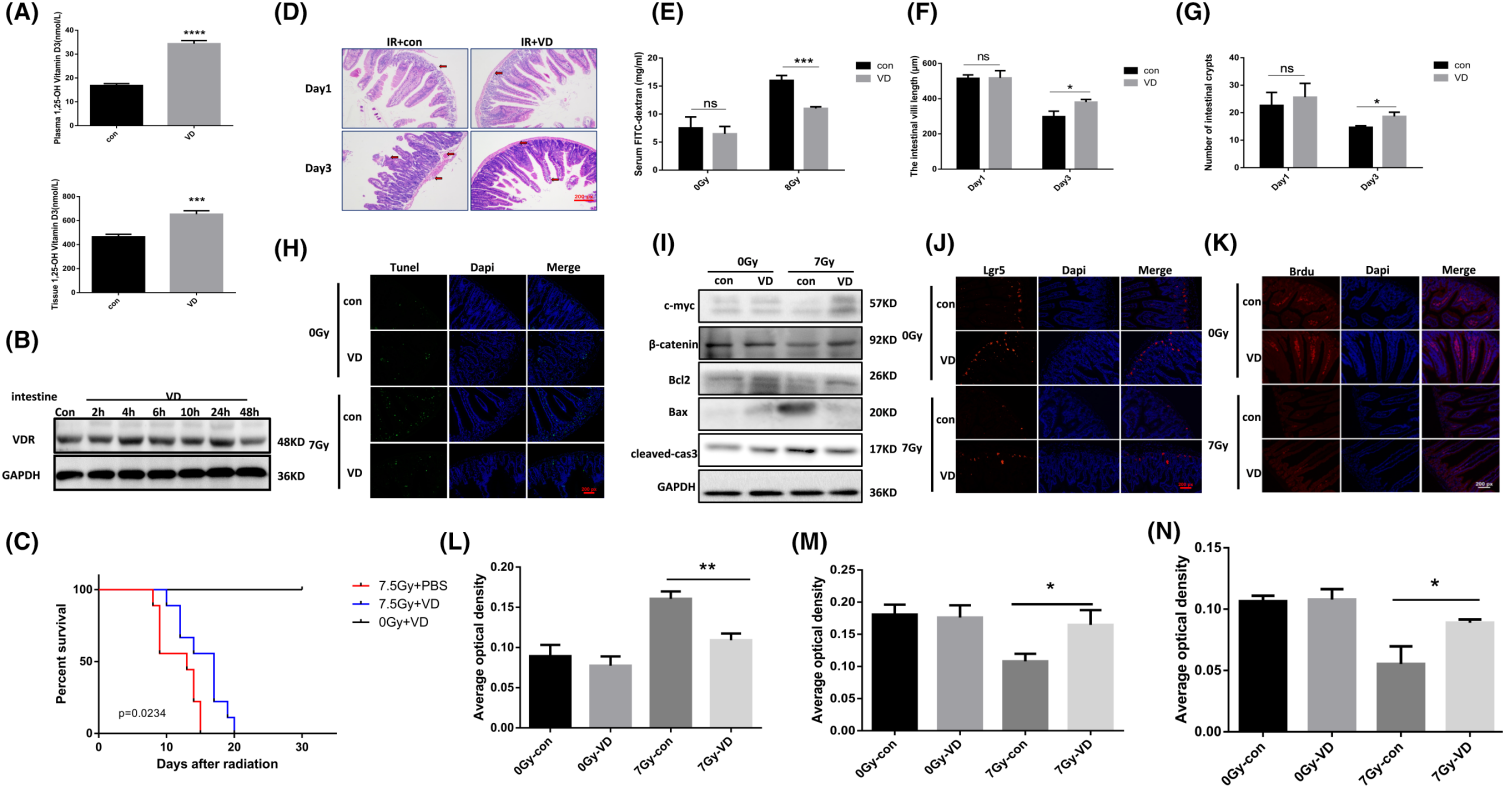
VDR agonists alleviate radiation‐induced intestinal damage in mice. (A) Effect of VDR agonist on 1,25‐(OH)2D metabolism in mice. (B) Effect of VDR agonist on VDR expression in small intestines of mice. (C) VDR agonist prolonged the average survival time of mice after irradiation. (D) H&E pathological test on mouse intestine post irradiation. (E) Expression level of FITC‐dextran in mouse serum. (F) The length of villi. (G) The number of intestinal crypts in mice. (H) The effect of VDR agonists on radiation‐induced apoptosis of intestinal epithelial cells was detected by Tunel immunofluorescence assay. (I) The role Calcitriol (VD) played on apoptosis of intestinal tissue after irradiation. (J) The effect of Calcitriol (VD) on Lgr5 + ISC post irradiation was detected by Lgr5+ mRNA fluorescence in situ hybridization (FISH). (K) The effect of Calcitriol (VD) on the proliferative capability of intestinal cells after irradiation was detected by the BrdU assay. (L) The bar chart depicting TUNEL staining. (M) The bar chart depicting Lgr5+ mRNA fluorescence in situ hybridization. (N) The bar chart depicting the BrdU staining. The statistical results are expressed by Mean ± SD, *, *** and **** represents *p* < 0.05, *p* < 0.001 and *p* < 0.0001, respectively. Each experiment was repeated for three times. *N* = 10

The experimental results showed that Calcitriol (VD), which was used as agonist of VDR in this study, can elevate the level of 1,25‐ (OH) 2D (Figure 2A) and activate the VDR expression in intestinal tissues (Figure 2B). Irradiated with a dose of 7.5 Gy (Figure 2C), Calcitriol (VD) prolonged the average survival time of mice post irradiation but exerted no positive effect on overall mortality. Pathology (Figure 2D,F,G) revealed that VDR agonists are able to attenuate radiation‐induced intestinal damage in mice. Due to the intestinal epithelial barrier, fluorescein isothiocyanate (FITC)‐dextran cannot enter the blood in normal intestinal tissues of mice. After ionizing radiation, the function of normal intestinal epithelial barrier is damaged, resulting in fluorescein isothiocyanate (FITC)‐dextran entering the blood via damaged barrier, which was detected by in vitro experiments. A prominent rise of fluorescein isothiocyanate (FITC)‐dextran was observed after irradiation, whose expression in the serum levels of mice can be inhibited by Calcitriol (VD) (Figure 2E), revealing the protective effect of the VDR agonist Calcitriol (VD) on radiation‐induced intestinal damage.

### 3.4 VDR agonists inhibit radiation‐induced intestinal epithelial cell apoptosis, attenuate radiation‐induced intestinal stem cell damage and promote intestinal epithelial cell repair after irradiation

The main form of cell death induced by radiation is apoptosis. The levels of apoptosis in intestinal cells also reflect the extent of intestinal damage after ionizing radiation. Experiments showed that Calcitriol (VD) can inhibit expression of Tunnel positive cells in intestinal epithelial tissues caused by irradiation (Figure 2H,L), which we hypothesized may be related to the ability of Calcitriol (VD) to alter the expression of Bax, cleaved‐caspase3 post irradiation (Figure 2I). The self‐renewal speed of intestinal tissues, especially intestinal stem cells in the crypts which are extremely important for intestinal tissue regeneration, is rapid and therefore sensitive to ionizing radiation. Lgr5+ intestinal stem cells, the most dominant population of intestinal stem cells, can differentiate into all intestinal epithelial cell groups, the staining of which therefore better reflects the number of intestinal stem cells. Lgr5 fluorescence in situ hybridization (Figure 2J,M) indicated that Calcitriol (VD) alleviates radiation‐induced intestinal stem cell damage. Detecting the intestinal epithelial proliferation function, BrdU immunofluorescence staining (Figure 2K,N) demonstrated that the proliferation capability of intestinal tissue cells post irradiation decreased, and that Calcitriol (VD) can promote the proliferation and repair of intestinal tissue cells after irradiation.

### 3.5 Vitamin D deficiency exacerbates radiation‐induced intestinal damage

**FIGURE 3:**
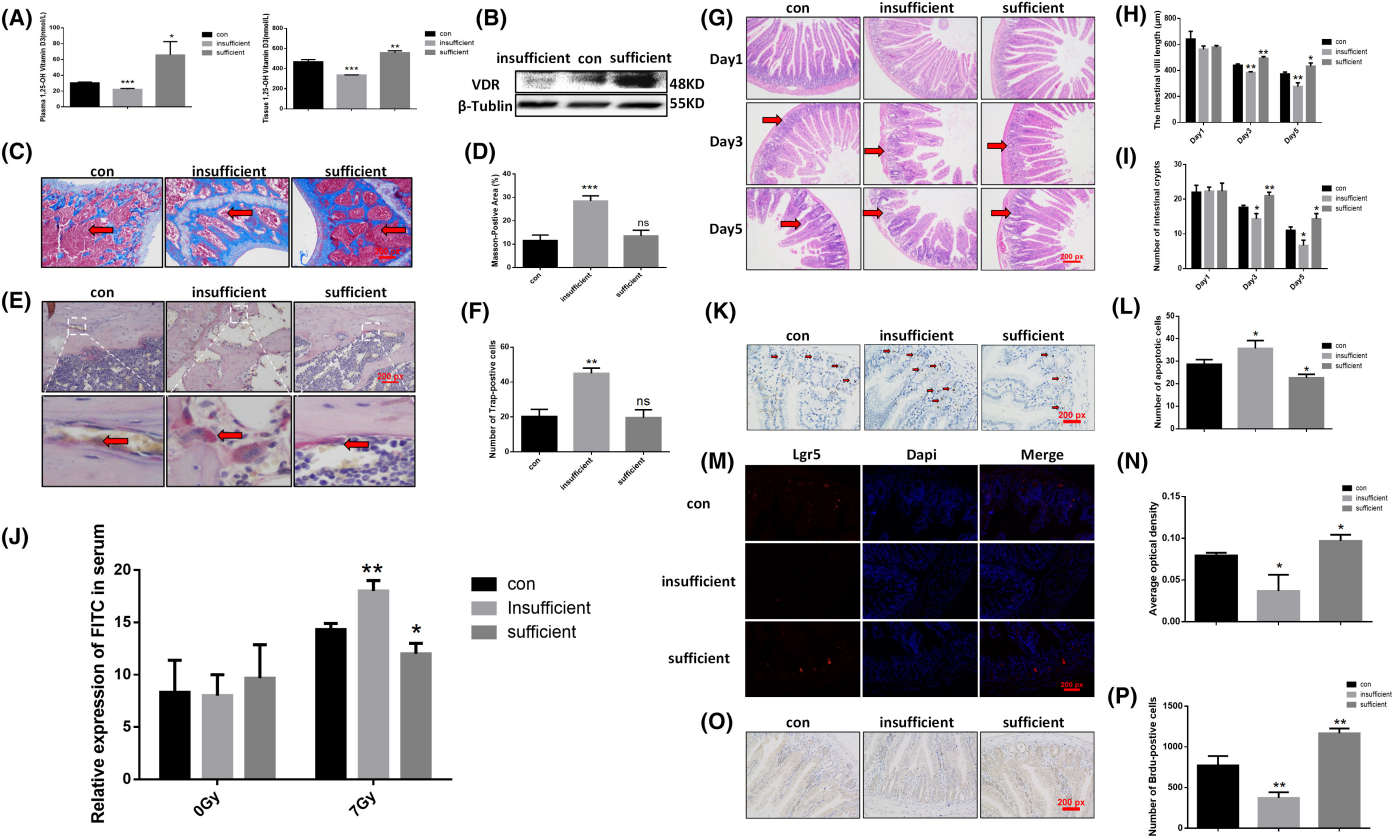
Vitamin D insufficient/sufficient mouse model was constructed and radiation‐induced intestinal injury was analysed. (A) The level of 1,25‐(OH)2D in serum and intestinal tissues of Vitamin D insufficient/sufficient mice was detected by ELISA. (B) Intestinal VDR expression of Vitamin D insufficient/sufficient mice were detected by the Western blot test. (C) Femurs of Vitamin D insufficient/sufficient mice were stained by Masson's trichrome. (D) The bar chart depicting Masson Staining. (E) TRAP staining of femurs of Vitamin D insufficient/sufficient mice. (F). statistical chart depicting TRAP staining. (J) FITC‐dextran level in serum of Vitamin D insufficient/sufficient mice before and after irradiation. (G) H&E pathological test on the intestine of Vitamin D insufficient/sufficient mice post irradiation. (H) The length of villi stained with H&E. (I) The intestinal crypt number stained with H&E. (K) Immunohistochemical staining of Vitamin D insufficient/sufficient mice post irradiation. (L) Bar chart depicting the Immunohistochemical staining. (M) Mouse intestinal tissue was detected by Lgr5+ mRNA fluorescence in situ hybridization on day 3 after irradiation. (N) The statistical chart of fluorescence in situ hybridization. (O) Intestinal tissue of Vitamin D insufficient/sufficient mice was detected on day 3 after irradiation by the BrdU assay. (P) The bar chart depicting the BrdU staining. The statistical results are expressed by Mean ± SD, *, **, and *** represents *p* < 0.05, *p* < 0.01 and *p* < 0.001, respectively. Each experiment was repeated for three times. *N* = 10

To validate the role of VDR in radiation‐induced intestinal damage, we constructed mouse model of vitamin D‐deficient mice and vitamin D‐high mice by dietary control. We verified the model by detecting the serum level of 1,25‐OH vitamin D (Figure 3A) and intestinal VDR expression (Figure 3B), and examining degree of osteoporosis by and Masson's Trichrome Stain (Figure 3C,D) and Trap Stain (Figure 3E,F) respectively. The results demonstrated that vitamin D‐deficient mice had a low serum level of 1,25‐OH vitamin D and subsequently an obvious osteoporosis. Vitamin D‐deficient/high mice were taken for irradiation and H&E staining, and results indicated that the vitamin D‐deficient group shortened intestinal villus length, deteriorated intervillous inflammatory exudation, triggered intestinal basal shedding, and reduced the number of crypts (Figure 3G–I). The intestinal apoptosis in vitamin D‐deficient mice increased after ionizing radiation, while the level in vitamin D‐high group decreased (Figure 3K,L). Fluorescein isothiocyanate (FITC) ‐dextran assay (Figure 3J) suggested that compared with control groups, the fluorescence intensity of vitamin D‐deficient mice serum increased, indicating that dietary deficiency of vitamin D aggravates the intestinal damage post irradiation. To explore the effect of vitamin D deficiency on the number of Lgr5+ intestinal stem cells and intestinal tissue proliferation after ionizing radiation, experimental results demonstrated that (Figure 3M–P) vitamin D deficient mice showed a further decrease in the number of Lgr5+ positive cells and the proliferative capability of small intestinal tissue post irradiation. The vitamin D‐high group, however, showed the opposite results, revealing that vitamin D is involved in the proliferation and repair of intestinal tissue after ionizing radiation. Lack of vitamin D can significantly inhibit the self‐repair of intestinal tissues probably on account of the increased radiosensitivity of intestinal stem cells ensuing vitamin D deficiency.

### 3.6 Effect of VDR agonists on the radiosensitivity of MODE‐K cells

**FIGURE 4:**
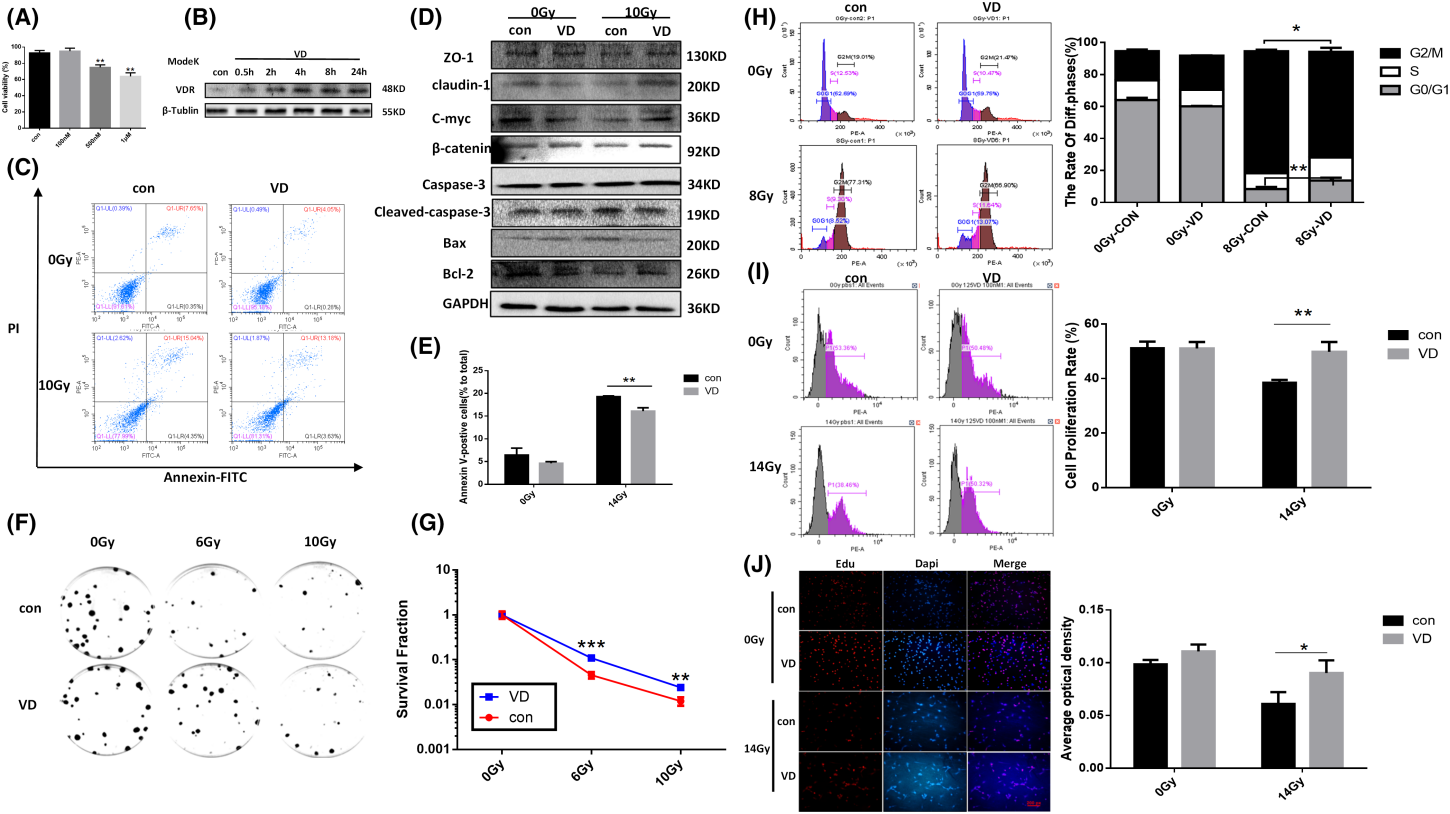
VDR agonists lowered the radiosensitivity of Modek cells. A. The optimal concentration of VDR agonists was screened by CCK8 assay. (B) Calcitriol (VD) induced VDR expression in intestinal epithelial cells. (C) The effect of Calcitriol (VD) on the intestinal epithelial cell apoptosis post irradiation was detected by flow cytometry. (D) Western blot was used to verify the effect of Calcitriol (VD) on the expression of apoptosis protein, barrier protein and prolife in in intestinal epithelial cells after irradiation was verified by Western Blot Analysis. (E) The bar chart depicting apoptosis. (F) The effect of Calcitriol (VD) on the proliferative capability of Modek cells post irradiation was detected by clone formation assay. (G) The statistical chart of clone formation assay. (H) The effect of Calcitriol (VD) on the cell cycle of Modek cells post irradiation was detected by flow cytometry. (I) The effect of Calcitriol (VD) on the proliferative capability of Modek cells post irradiation was detected through Edu assay by flow cytometry. (J) The effect of Calcitriol (VD) on the proliferative capability of Modek cells post irradiation was detected through Edu assay by immunofluorescence. The statistical results are expressed by Mean ± SD, *, **, and *** represents *p* < 0.05, *p* < 0.01 and *p* < 0.001, respectively. Each experiment was repeated for three times.

Firstly, Calcitriol (VD) at a dose of 100 nM (Figure 4A) was able to upregulate VDR expression in MODE‐K cells, which was time‐dependent, with the highest expression at 24 h (Figure 4B). On this basis, via irradiation to MODE‐K cells, Calcitriol (VD) was found to inhibit radiation‐induced apoptosis in MODE‐K cells (Figure 4C,E). Further detecting expression of relevant proteins (Figure 4D), we found that Calcitriol (VD) can alleviate the radiation inhibition of Bcl2, β‐catenin, c‐myc, ZO‐1 and claudin1, and simultaneously downregulate the radiation expression of Bax, cleaved‐cas3 (cleaved‐caspase3). Then the proliferative capacity of intestinal cells post irradiation was further examined. First of all, the clonogenic assay (Figure 4F,G) found that Calcitriol (VD) attenuates radiosensitivity of MODE‐K cells, increases the number of cell colonies post irradiation and promotes cell survival after irradiation. Moreover, cell cycle assay (Figure 4H) found that ionizing radiation can prominently aggravate the G2M phase arrest in MODE‐K cells, compared with Calcitriol (VD) which inhibits the radiation‐induced G2M phase arrest. Furthermore, detecting the proliferative capability of MODE‐K cells after 14 Gy irradiation via flow cytometry and immunofluorescence assay (Figure 4I), we found that Calcitriol (VD) can alleviate the radiation inhibition of cell proliferation peaks. The results of immunofluorescence assay (Figure 4J) were similar that Calcitriol (VD) can upregulate the fluorescence intensity of Edu‐positive cells post irradiation. Calcitriol (VD) is able to promote cells to go through the cycle checkpoints after radiation, enabling MODE‐K cells to split and proliferate to repair radiation‐induced intestinal epithelial cell damage, which is important for intestinal epithelial barrier function.

### 3.7 Knockdown/overexpression of VDR exerted opposite damaging effects on MODE‐K cells after radiation

**FIGURE 5:**
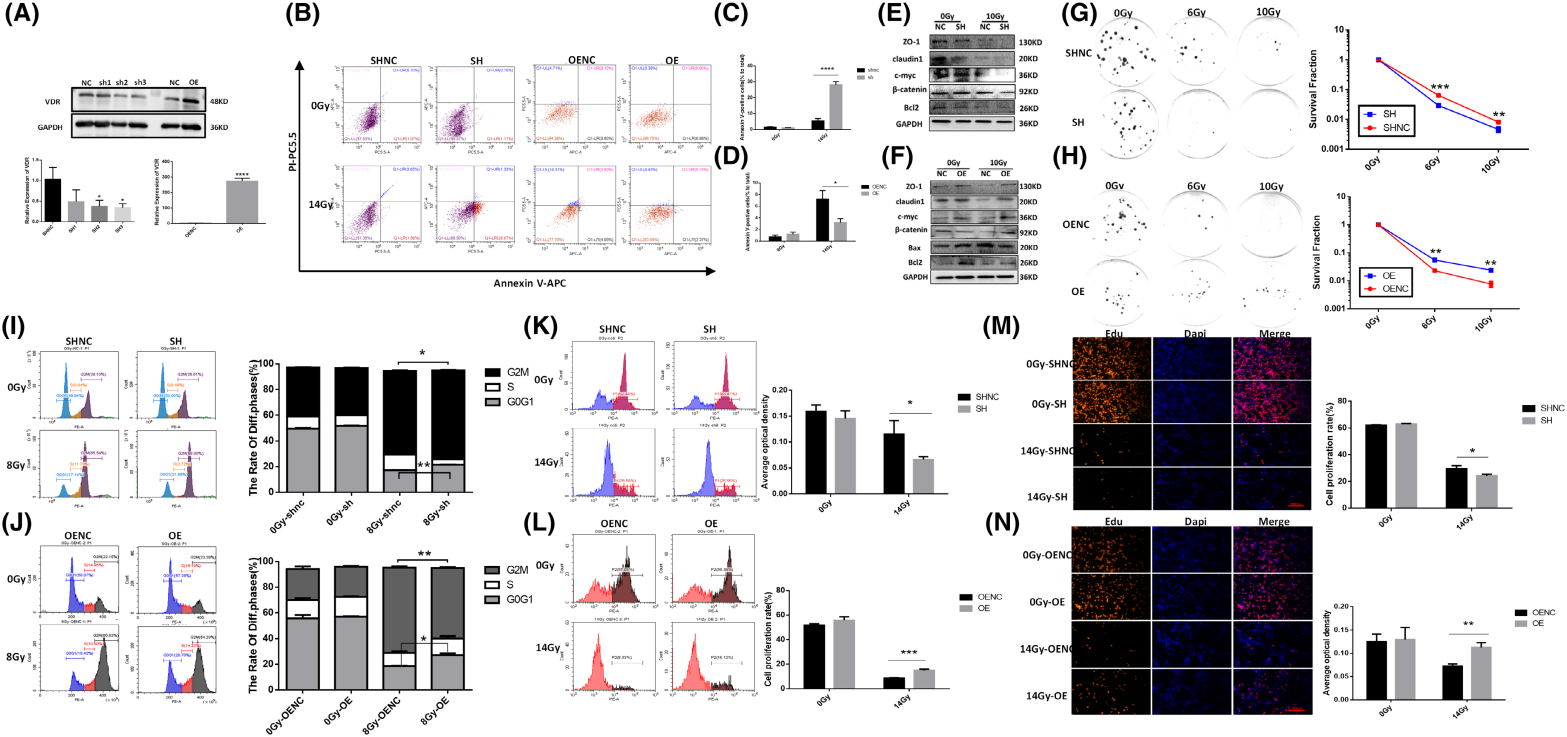
The effect of overexpression/knockdown of VDR on the radiosensitivity of Modek cells. (A) The expression level of VDR in Modek cell lines was verified by Western blot/RT‐QPCR test. (B) The effect of knockdown/overexpression of VDR on intestinal epithelial cell apoptosis after irradiation was detected by flow cytometry assay. (C) The statistical chart depicting the effect of VDR knockdown on cell apoptosis after irradiation. (D) The statistical chart depicting the effect of VDR overexpression on cell apoptosis after irradiation. (E) The effect of VDR knockdown on the expression of corresponding proteins in intestinal epithelial cells after irradiation was verified by Western blot Analysis. (F) The effect of VDR overexpression on the expression of corresponding proteins in intestinal epithelial cells after irradiation was verified by Western blot Analysis. (G) The effect of VDR knockdown on the proliferative capability of Modek cells post irradiation was detected by clone formation assay. (H) The effect of VDR overexpression on the proliferative capability of Modek cells post irradiation was detected by clone formation assay. (I) The effect of VDR knockdown on the cell cycle of Modek cells post irradiation was detected by flow cytometry. (J) The effect of VDR overexpression on the cell cycle of Modek cells post irradiation was detected by flow cytometry. (K) The effect of VDR knockdown on the proliferative capability of Modek cells post irradiation was detected through Edu assay by flow cytometry. (L) The effect of VDR overexpression on the proliferative capability of Modek cells post irradiation was detected through Edu assay by flow cytometry. (M) The effect of VDR knockdown on the proliferative capability of Modek cells post irradiation was detected through Edu assay by immunofluorescence. (N) The effect of VDR overexpression on the proliferative capability of Modek cells post irradiation was detected through Edu assay by immunofluorescence. The statistical results are expressed by Mean ± SD, *, **, and *** represents *p* < 0.05, *p* < 0.01 and *p* < 0.001, respectively. Each experiment was repeated for three times.

Modek‐VDR‐SH and Modek‐VDR‐OE cell lines were constructed using the plasmids. Western Blot and RT‐qPCR (Figure 5A) were conducted for validation. To further verify the effect of knockdown/overexpression of VDR on the apoptosis level of MODE‐K cells after radiation, the results showed (Figure 5B–D) that VDR knockdown aggravated radiation‐induced apoptosis, while VDR overexpression inhibited radiation‐induced apoptosis. Knockdown of the VDR can intensify the radiation inhibition of bcl2, −catenin, c‐myc, ZO‐1 and claudin1. After VDR overexpression, however, it was found that protein expression was opposite to the effect of VDR knockdown (Figure 5E,F), revealing that VDR can reduce radiation‐induced intestinal epithelial cell damage, promote intestinal epithelial cell proliferation after radiation, and further play an important role in the maintenance of intestinal epithelial barrier function.

### 3.8 Knockdown of VDR further aggravated the radiation inhibition of MODE‐K cell proliferation, opposite to the effect of VDR overexpression

Clonogenesis assay revealed that radiation resulted in a decrease in the number of cell clones, and knockdown of VDR (SH) further enhanced the radiosensitivity of cells (Figure 5G), but overexpression of VDR (OE) increased cell clones (Figure 5H). Knockdown of VDR further aggravated the radiation‐induced G2‐M arrest (Figure 5I), decreased the proliferative capability (Figure 5K,M) of Modek cells. With the subsequent flow cytometry and fluorescence microscopy tests, we found that the proliferative capability of MODE‐K cells was diminished after VDR knockdown, which is also well correlated with previous experimental findings. Further examining the cellular effects after VDR overexpression (Figure 5J,L,N), we found that overexpression of VDR alleviated the radiation inhibition of MODE‐K cell proliferation. As is concluded, radiosensitivity of MODE‐K cells increased upon knockdown of VDR but decreased after VDR overexpression.

### 3.9 VDR agonists reduce intestinal stem cell damage and promote proliferation and differentiation post irradiation

**FIGURE 6:**
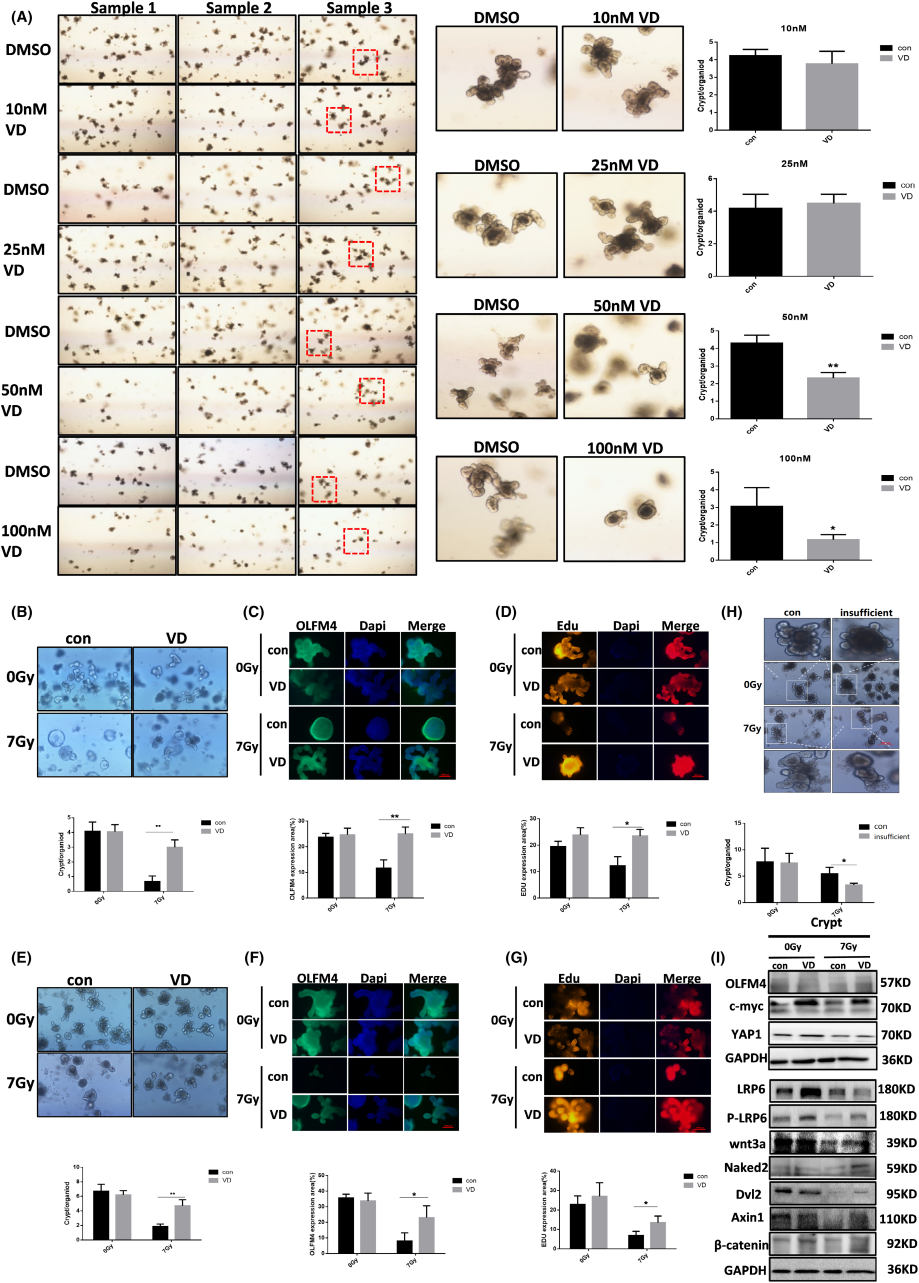
Effect of VDR on the function of intestinal stem cells. (A) Drug toxicity testing on intestinal organoids. In vivo experiments: (B) Intestinal organoid culture. (C) Immunofluorescence staining of OLFM4. (D) Organoid proliferation was detected through Edu assay by immunofluorescence. In vitro experiments: (E) Intestinal organoid culture. (F) Immunofluorescence staining of OLFM4. (G) Organoid proliferation was detected through Edu assay by immunofluorescence. (H) Vitamin D deficiency aggravates damage to radiation‐induced intestinal organoids. (I) VDR agonists regulates Wnt pathway in intestinal epithelial cells after irradiation. The statistical results are expressed by Mean ± SD, *, and ** represents *p* < 0.05 and *p* < 0.01, respectively. Each experiment was repeated for three times.

First, we found that the optimal drug dose of Calcitriol (VD) (Figure 6A) in vitro experiments on mouse intestinal organoids was 25 nM. In vivo and in vitro experiments indicated that γ‐ray irradiation at a dose of 7 Gy significantly caused damage to intestinal organoids, with organoids expansion and even rupture, reduced budding, and weakened differentiation function (Figure 6B,E). Nevertheless, Calcitriol (VD) can reduce radiation‐induced damage to intestinal organoids and alleviate the radiation inhibition of fluorescence expression of OLFM4 (Figure 6C,F) and EDU (Figure 6D,G), suggesting that Calcitriol (VD) attenuates radiation‐induced ISC damage and promotes the proliferative repair of ISC. In consistent with above results, deficiency of Vitamin D aggravated the damage of radiation to intestinal organoids (Figure 6H). To further validate the molecular role of Calcitriol (VD) on ISC regulation after irradiation, we found in the results of the Western blot experiment (Figure 6I) that Calcitriol (VD) can promote the expression of OLFM4, YAP1 and c‐myc post irradiation. Further detection of the Wnt pathway revealed that Calcitriol (VD) alleviated wnt3a inhibition after ionizing radiation, accompanied with the altered expression of LRP6, P‐LRP6, Naked2, Dvl2, and Axin1. Finally, through irradiation of intestinal organoids of mice lacking in vitamin D, it was found that the radiosensitivity of intestinal organoids was upregulated after vitamin D deficiency, manifested by decreased budding and rupture of intestinal organoids after radiation.

### 3.10 VDR regulates the radiosensitivity of intestinal epithelial cells by targeting HIF/PDK1

**FIGURE 7:**
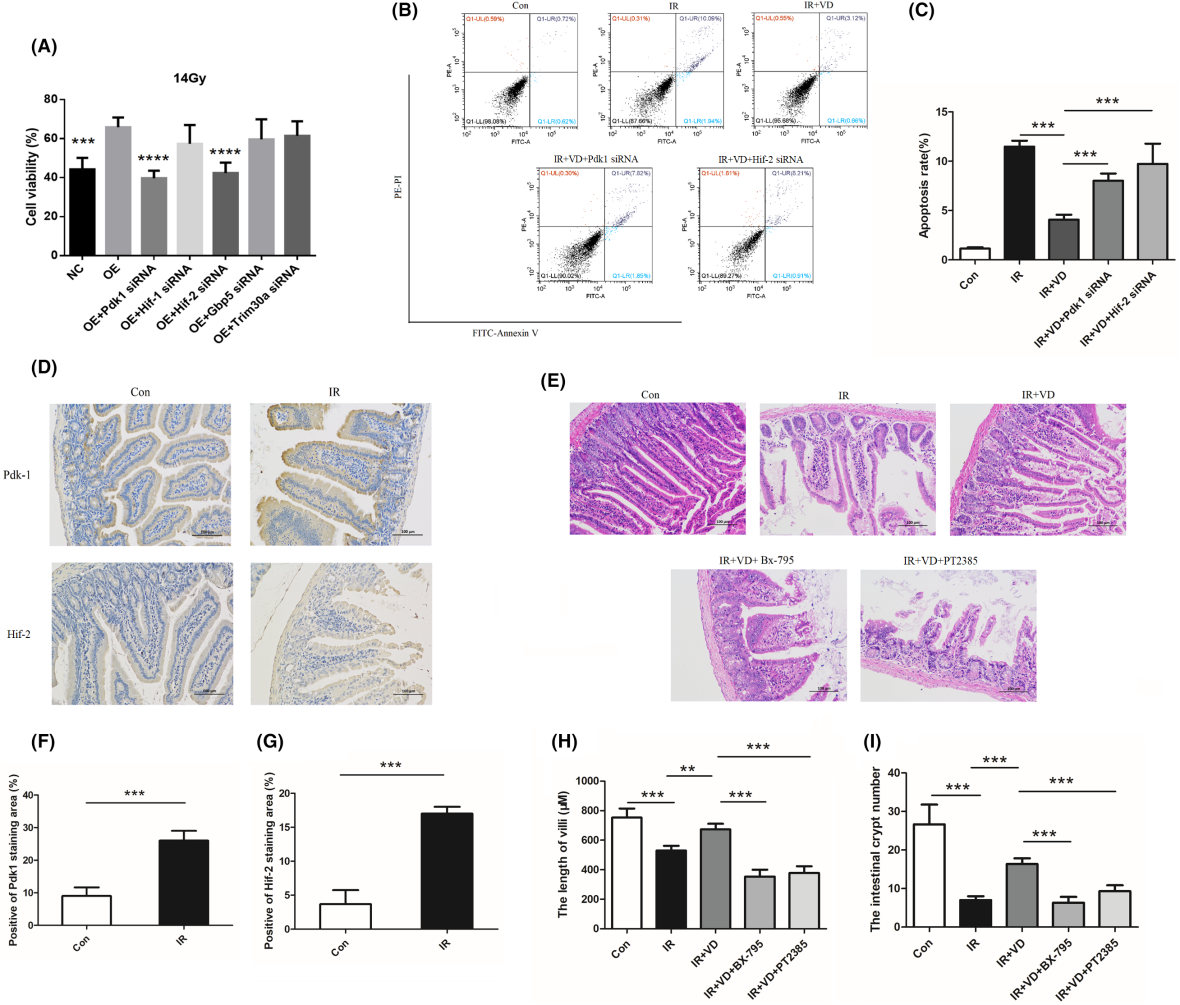
VDR reduced the radiosensitivity of intestinal epithelial cells and alleviated radiation‐induced intestinal damage by targeting HIF/PDK1. (A) CCK8 assay verified the effect of each siRNA on the viability of Modek‐OE cells after irradiation. (B) With Pdk1 and Hif‐2 siRNA, the apoptosis was detected after irradiation and VDR agonist, 1,25‐(OH)2D using. (C) The quantity analysis for apoptosis in radiated Modek cells. (D) 3 days after irradiation, the expression of PDK1 and HIF‐2 were tested by immunohistochemical staining and were quantified (F, G). (E) Mice were intraperitoneal injected with Bx‐795 (inhibitor of PDK1) at 5 mg/kg and PT2385 (inhibitor of HIF2) at 10 mg/kg before irradiation and 1,25‐(OH)2D using, and 3 days later, the small intestine was taken to perform H&E staining, and the length of villi (H) and number of intestinal crypt (I) were quantified to indicate the degree of RIII. The statistical results are expressed by Mean ± SD, **, and *** represents *p* < 0.01 and *p* < 0.001, respectively. Each experiment was repeated for three times.

To further investigate the mechanism of VDR in radiation‐induced intestinal damage, Modek‐NC and Modek‐SH cell lines went through differential gene screening by means of transcriptome sequencing. Differential genes screening between groups was based on the significant level of *p*<0.05 and fold change of |log 2FC|>1. A total of 390 differential genes were caused by VDR knockdown, in which 232 genes were downregulated and 158 genes were upregulated, as compared with the NC group (Figure S1A–H). Gene ontology (GO) analysis and KEGG pathway analysis showed that the differential genes caused by VDR knockdown are mostly involved in cellular processes of defence response to virus, and cellular response to interferon‐beta, and related with cell growth and death, transport and catabolism (Figure S1I–J). Clustering analysis followed (Figure S1K) with PDK1, GBP5, TRIM30a, NOTCH4 and Rsad2 used as central molecules. After RNA was extracted through the TRIZOL assay, each gene was validated using RT‐qPCR. Results (Figure S1L) indicated that after knockdown of VDR, VDR expression in intestinal epithelial cells was downregulated, accompanied by the downregulation of expression of HIF1, HIF2, PDK1, GBP5, Trim30a, Rsad2, and notch4. Subsequently we further constructed the siRNA (small interfering RNA) of PDK1, GBP5, and TRIM30. After γ‐ray irradiation at a dose of 14 Gy, we found that (Figure 7A), VDR overexpression can upregulate the viability of Modek cells after irradiation. Compared with the overexpressing VDR (OE) group, the cell viability of MODE‐K cells caused by VDR overexpression post irradiation can be inhibited by interfering PDK1 and HIF2, suggesting that VDR may exert protective effects on radiation‐induced intestinal epithelial damage by targeting PDK1 and HIF2. In addition, interfering PDK1 and HIF2 by siRNA, all attenuated the anti‐apoptotic effect of VD (Figure 7B,C).

Further, the role of PDK1 and HIF2 were also investigated in vivo. Firstly, the expressions of PDK1 and HIF2 in small intestine tissues were detected after irradiation by immunohistochemical staining, and results demonstrated that irradiation up‐regulated the expression of PDK1 and HIF2 (Figure 7D,F,G). Next, using the BX‐795 and PT2385 to inhibit PDK1 and HIF2 respectively, the damage in radiated small intestine was more severe than the VD group (Figure 7E,H,I), which indicated that PDK1 and HIF2 mediate the protective effect of VD on the RIII.

## 4 DISCUSSION

Given that there are no effective ways for prevention and treatment targeted at radiation‐induced intestinal injury which is a major dose‐limiting factor for abdominal and pelvic radiotherapy, it is of great significance in the realm of ionizing radiation to find a protective target to resolve radiation‐induced intestinal damage at this stage.

In our study, we found that both whole body and local abdominal irradiation could activate VDR of small intestinal tissue in mice. Actually, despite few reports about the relationship between ionizing radiation and VDR, it has been clarified that UV irradiation promotes skin vitamin D synthesis.^14^, ^15^, ^16^ Ionizing radiation can directly induce increased VDR expression in MODE‐K cells, which is dose and time‐dependent. It should be illustrated that, clear as the direct effect has been, the potential indirect effect cannot be denied. In light of the body sophistication, more experiments are required for the clarification of this subject. Besides illuminating the effect of ionizing radiation on intestinal VDR expression, we also found a negative correlation between VDR expression post radiation and intestinal damage, which seems inconsistent with the conclusion that irradiation induces VDR expression. In general, irradiation triggers intestinal damage and VDR expression simultaneously, both presenting a positive correlation. The paradox in the experimental results, however, just suggests that the increased VDR expression induced by irradiation may be a protective mechanism provoked by systemic stress response, which exists extensively from a biological standpoint.

In the following experiments, we investigated the specific role of VDR in radiation‐induced intestinal injury in mice. It was found that VDR agonists can alleviate radiation‐induced intestinal epithelial tissue damage and relieve the intestinal epithelial barrier dysfunction, which may be accomplished by VDR involved in the regulation of apoptotic proteins post irradiation and promotion of cell proliferation. Meanwhile, we also found that VDR agonists attenuate radiation‐induced intestinal stem cell damage, exerting crucial effects on intestinal epithelial differentiation. In the vitamin D deficiency and high vitamin D mouse models constructed by dietary control of vitamin D intake, the mouse intestinal tissue was found increasingly sensitive to irradiation after vitamin D deficiency, further illustrating the involvement of VDR in the damage repair of the intestinal epithelium.

The killing effect of ionizing radiation on intestinal epithelial cells which serve as a vital component of the intestinal epithelial barrier^17^, ^18^ manifests itself in direct damage and indirect damage caused by hydroxyl radicals and reactive oxygen species from the ionized cellular and tissue moisture.^5^, ^19^ DNA damage in intestinal epithelial cells is mainly manifested by DNA fracture, suppressed cell proliferation, cycle arrest and increased apoptosis rate.^20^ Intestinal epithelial barrier damage^21^ is usually accompanied by fluid loss, infection and bacterial translocation, producing pernicious influence on the prognosis of radiotherapy.^22^, ^23^ Radiosensitivity of the intestinal epithelial cells was found to decrease after VDR was agonized, which may be related to the increased anti‐injury, proliferative and repair capability post irradiation. Ensuing further knockdown of VDR, we found that the radiosensitivity of intestinal epithelial cells increased, which is an adverse event for intestinal epithelial cells, aggravating the cell damage after radiation and weakening the cell damage repair capability. After overexpression of VDR, however, the radiation‐induced cellular biological effects were opposite to that caused by the knockdown of VDR. It has been illustrated in some studies that intestinal epithelial VDR knockout mice have more severe colitis than wild‐type mice in TNBS‐induced colitis models. Loss of VDR expression in intestinal stem cells leads to intestinal stem cell damage, which may be associated with interfered WNT signalling in intestinal stem cells and intestinal villi by low VDR expression.^24^, ^25^ Through in vitro and in vivo culture experiments on irradiated organoids, we found that VDR agonists can elevate the post irradiation survival rate of intestinal organoids and promote budding differentiation. These effects may be achieved by VDR agonists regulating β‐catenin expression by means of altering wnt3a pathway‐related proteins after irradiation to trigger c‐myc expression, which exerts protective effects on intestinal stem cells. This is consistent with the effect where agonized VDR promotes the proliferation of organoids after irradiation. Besides, we found that the radiosensitivity of vitamin D‐deficiency intestinal organoids increased.

Moreover, we further investigated the mechanism of VDR in regulating radiosensitivity of intestinal epithelial cells. Firstly, Modek cell lines knockout of VDR went through transcriptomic sequencing, from which a series of differential genes was screened by RNA‐SEQ and subsequently validated by RT‐qPCR. The differential genes regulated by VDR were screened through siRNA and eventually selected, indicating that interfering with PDK1 and HIF2 can reverse the effect caused by VDR overexpression. Therefore, we hypothesized that VDR may target the HIF/PDK1 pathway to alleviate radiation‐induced intestinal damage, providing new ideas for the prevention of radiation‐induced intestinal injury.

## 5 CONCLUSION

This study demonstrates that VDR may target the HIF/PDK1 pathway to attenuate radiation‐induced intestinal damage in mice and promote the repair of epithelial damage in intestinal stem cells, suggesting that the protective effect long‐term intake of vitamin D may have against radiation‐induced intestinal damage.

## AUTHOR CONTRIBUTIONS

**Penglin Xia:** Investigation (equal). **Cheng Zhang:** Investigation (equal). **Yajie Yang:** Investigation (equal). **Haishan Lin:** Writing – original draft (equal). **Hu Liu:** Resources (equal). **Ruling Liu:** Methodology (equal); writing – original draft (equal). **Xiaodong Liu:** Supervision (equal). **Jianming Cai:** Writing – review and editing (equal).

## FUNDING INFORMATION

This study was supported by National Natural Science Foundation of China (No. 81972978 and No. 81903260), the start‐up fund from the Beijing Natural Science Foundation (No.7214220 to H.L), the Friendship Seed Project (YYZZ202034 to H.L), Natural Science and Technology Foundation of Zunyi City (Grant No. (2019) 233).

## CONFLICT OF INTERESTS

The authors have no potential conflict of interests.

## CONSENT FOR PUBLICATION

All authors reached an agreement to publish the study in this journal.

## Supporting information


Supinfo1

Supinfo2

## Data Availability

All data generated or analysed during this study were included in this published article and its supplemental material.
