# Supplementary figures and images for: Protective effects of activated vitamin D receptor on radiation‐induced intestinal injury
Source: J Cell Mol Med. 2022 Dec 29;27(2):246–58. doi: 10.1111/jcmm.17645 (PMC9843524; doi:10.1111/jcmm.17645)

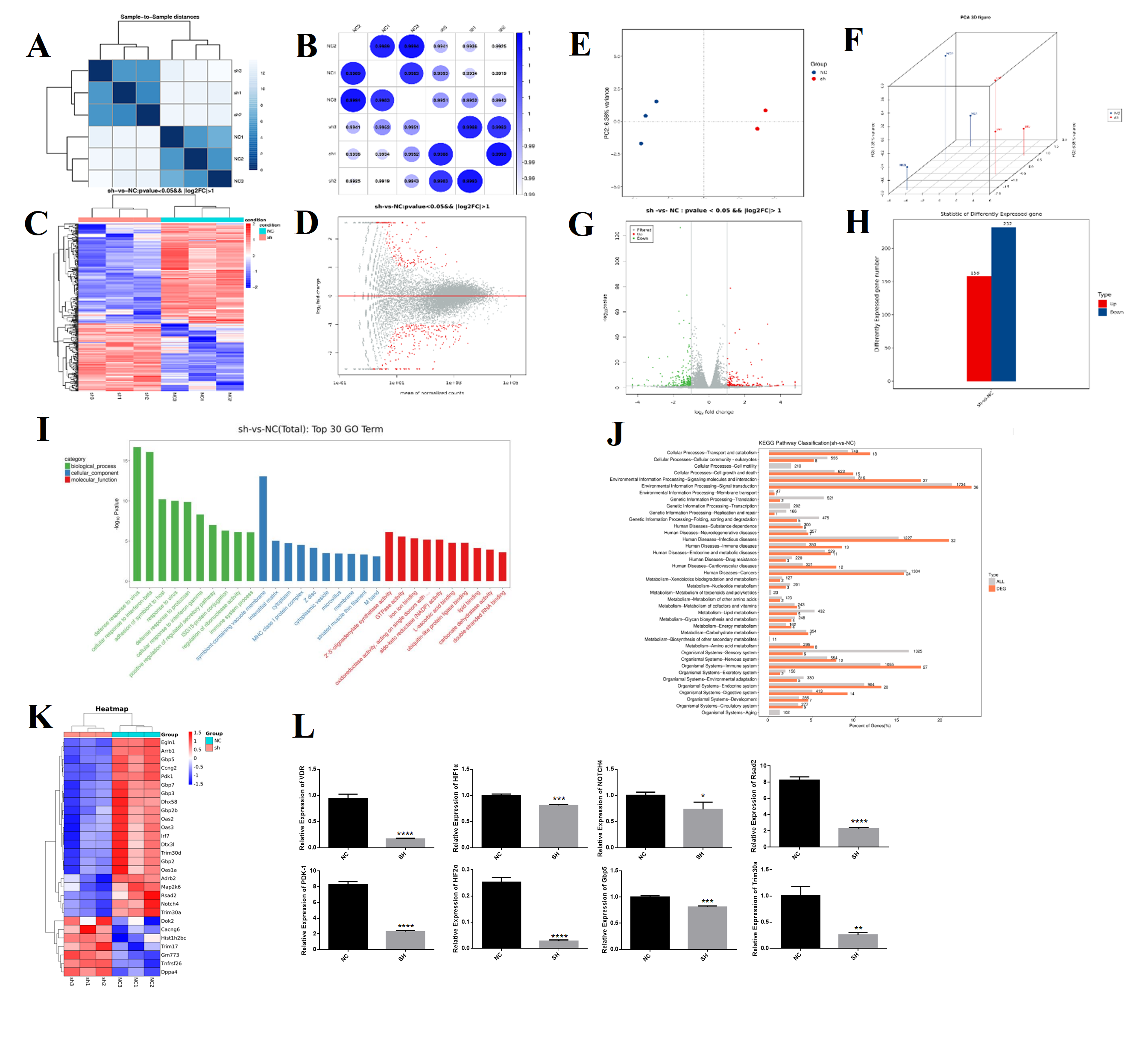

Supplement: Supplementary file 1 — Supinfo1 [file JCMM-27-246-s002.tif]
