# Supplementary material for: Protective effects of activated vitamin D receptor on radiation‐induced intestinal injury
Source: J Cell Mol Med. 2022 Dec 29;27(2):246–58. doi: 10.1111/jcmm.17645 (PMC9843524; doi:10.1111/jcmm.17645)
Supplement: Supplementary file 2 — Supinfo2 [file JCMM-27-246-s001.docx]

Supplemental material

Figure S1: Legends

A. Sample-to-sample cluster analysis results. B. Heat map of correlation coefficient between samples. C. PCA plot. D. PCA plot. E. Cluster analysis results of difference groups. F. MA plot for differentially expressed genes. G. Volcano plot for differentially expressed genes. H. Histogram of differentially expressed genes. I. GO analysis of differentially expressed genes. J. KEGG pathway analysis of differentially expressed genes. K. Heat map of differentially expressed genes. L. The expression of objective genes by RT-PCR. The statistical results are expressed by Mean ± SD, **, and *** represents P < 0.01 and P < 0.001, respectively. Each experiment was repeated for three times.
